# Supplementary material for: Electrolytes with Micelle-Assisted Formation of Directional Ion Transport Channels for Aqueous Rechargeable Batteries with Impressive Performance
Source: Nanomaterials (Basel). 2022 Jun 4;12(11):1920. doi: 10.3390/nano12111920 (PMC9182126; doi:10.3390/nano12111920)
Supplement: Supplementary file 1 [file nanomaterials-12-01920-s001.zip › nanomaterials-1736625-supplementary.pdf]

nanomaterials

Supplementary Material

Electrolytes with Micelle-Assisted Formation of Directional Ion Transport Channels for Aqueous Rechargeable Batteries with Impressive Performance

Yanmin Lu [1], Fengxiang Zhang [1], Xifeng Lu [2], Haihui Jiang [1], Wei Hu [1,*], Libin Liu [1] and Ligang Gai [1,*]

[1] Engineering & Technology Center of Electrochemistry, School of Chemistry and Chemical Engineering, Qilu University of Technology (Shandong Academy of Sciences), Jinan 250353, Shandong, China; 17854116233@163.com (Y.L.); 1043119202@stu.qlu.edu.cn (F.Z.); jhh@qlu.edu.cn (H.J.); lbliu@qlu.edu.cn (L.L.)
[2] School of Energy Materials, Shandong Polytechnic College, Jining 172000, Shandong, China; lxf-1979@126.com
* Correspondence: weihu@qlu.edu.cn (W.H.); liganggai@qlu.edu.cn (L.G.)

S1. Nyquist plots and theoretical computation results of LN-SDS-n

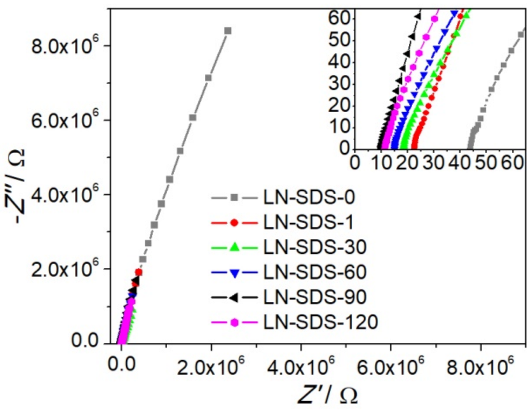

Figure S1. Room-temperature Nyquist plots of the electrolytes**,** which were filled with CR927 cells (Shenzhen Tianchenghe Science & Technology Ltd., Shenzhen, China) with a thickness of 0.26 cm and a radius of 0.3 cm.

Before electrochemical impedance spectra (EIS) measurements, the cells were placed at ambient temperature for 4 h, to allow sufficient contact between the electrolyte and the cell case. The ionic conductivity was calculated according to Equation (1), expressed as:

$$\sigma = L/(RS) \quad (1)$$

where L is the cell thickness, S is the contact area between the electrolyte and the cell case, and R is the electrolyte resistance represented by the x-intercept of the Nyquist plot.

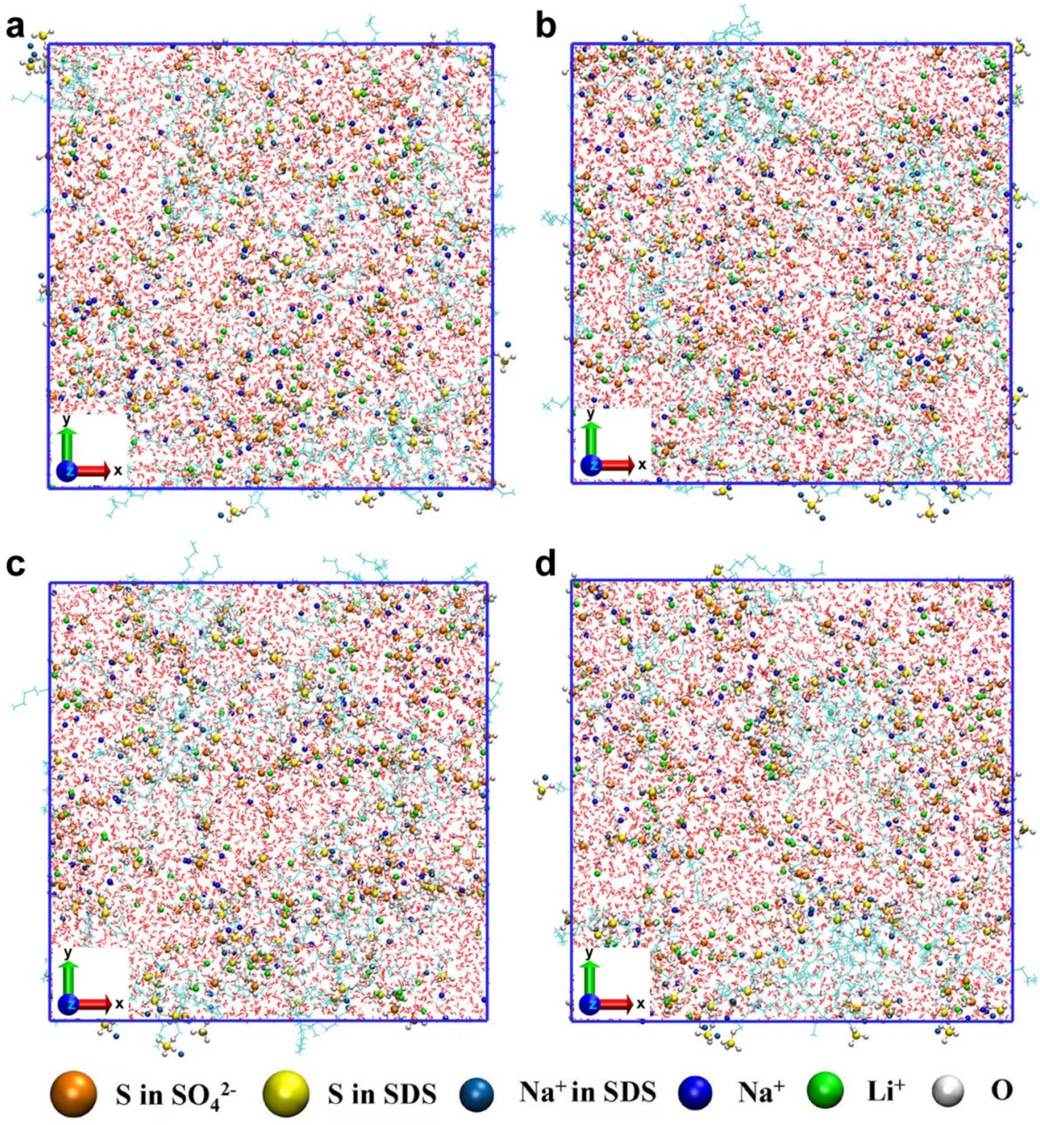

Figure S2. Molecular dynamic simulation results of LN-SDS-90 without an electric field at: (**a**) 258.15 K, (**b**) 263.15 K, (**c**) 268.15 K, and (**d**) 273.15 K. The small red molecules and the large cyan molecules are water and SDS paraffin chains, respectively.

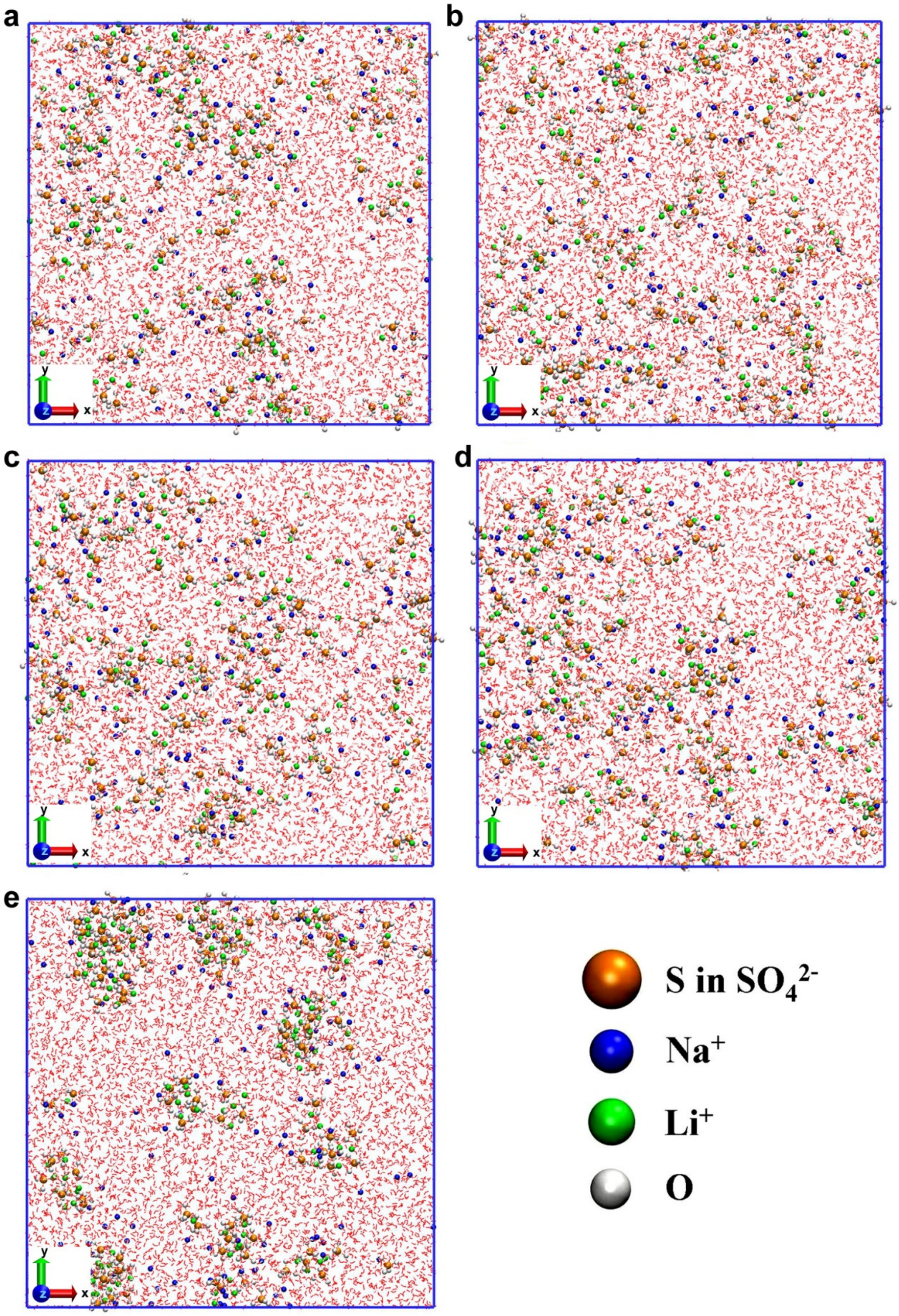

Figure S3. Molecular dynamic simulation results of LN-SDS-0 without an electric field at: (**a**) 258.15 K, (**b**) 263.15 K, (**c**) 268.15 K, (**d**) 273.15 K, and (**e**) 298.15 K.

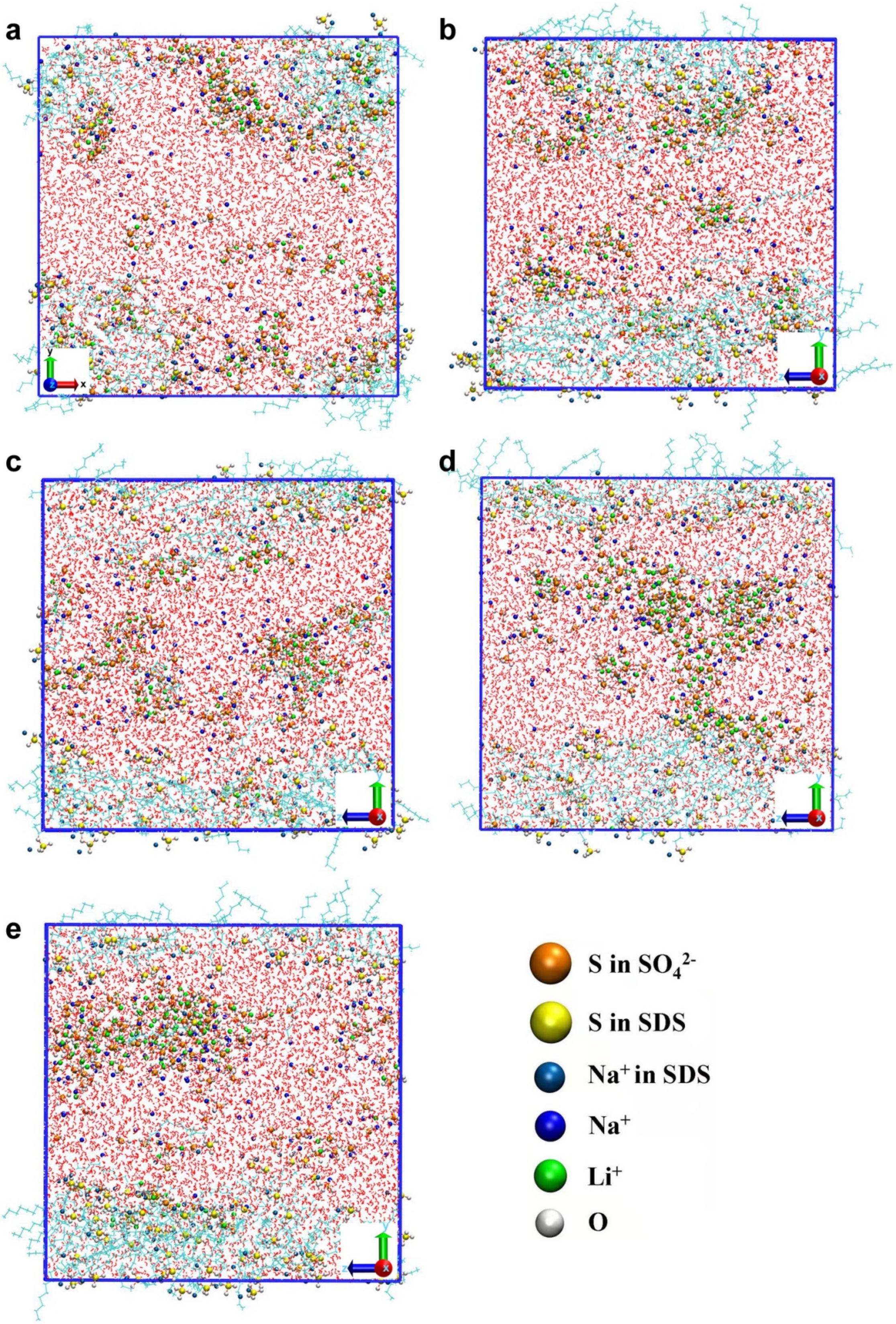

Figure S4. Molecular dynamic simulation results of room-temperature LN-SDS-90 without (**a**) and with applying voltages of: (**b**) 0.5 V nm^{-1}, (**c**) 1.0 V nm^{-1}, (**d**) 1.5 V nm^{-1}, and (**e**) 2.0 V nm^{-1}.

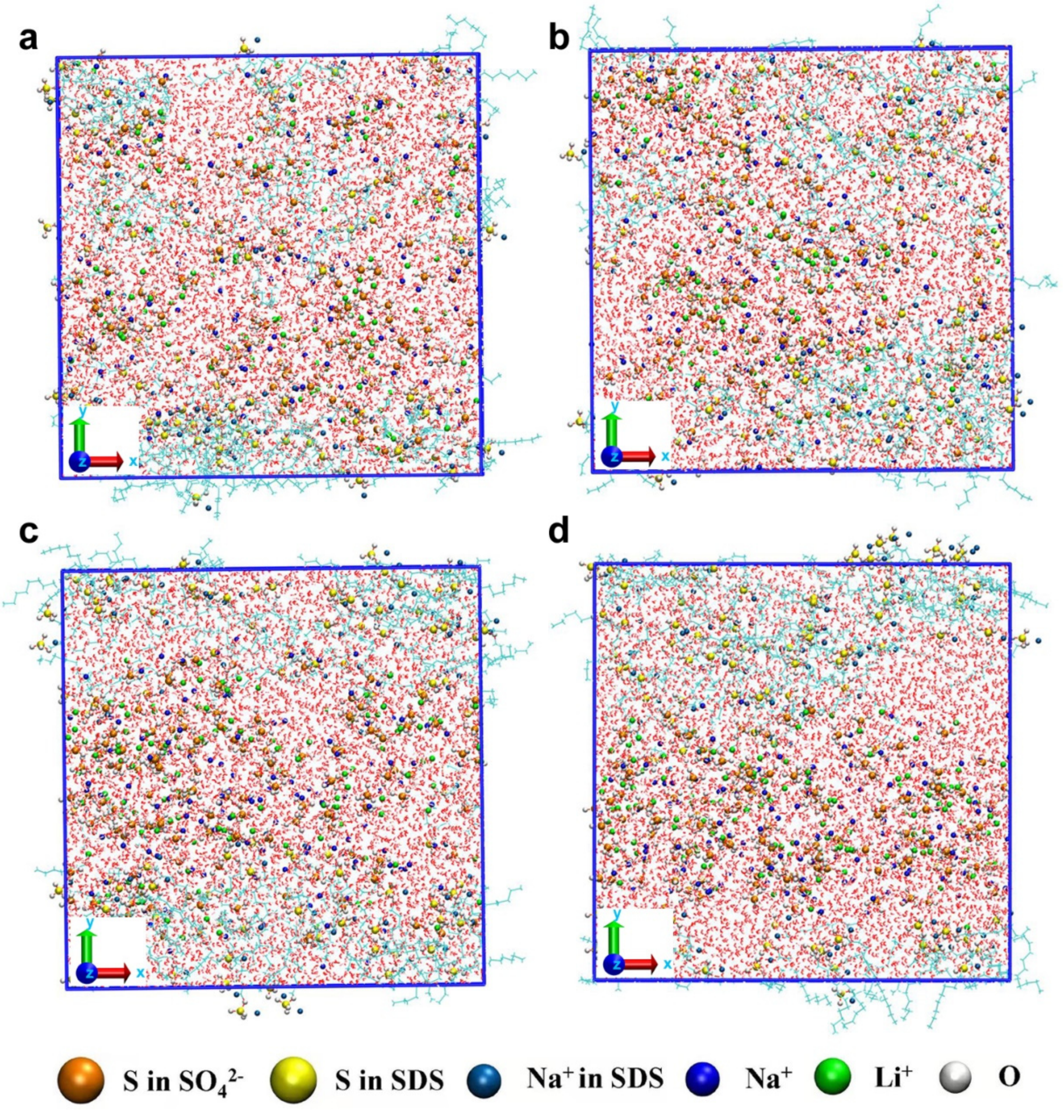

Figure S5. Molecular dynamic simulation results of LN-SDS-90 at 258.15 K, applying voltages of: (**a**) 0.5 V nm^{-1}, (**b**) 1.0 V nm^{-1}, (**c**) 1.5 V nm^{-1}, and (**d**) 2.0 V nm^{-1}.

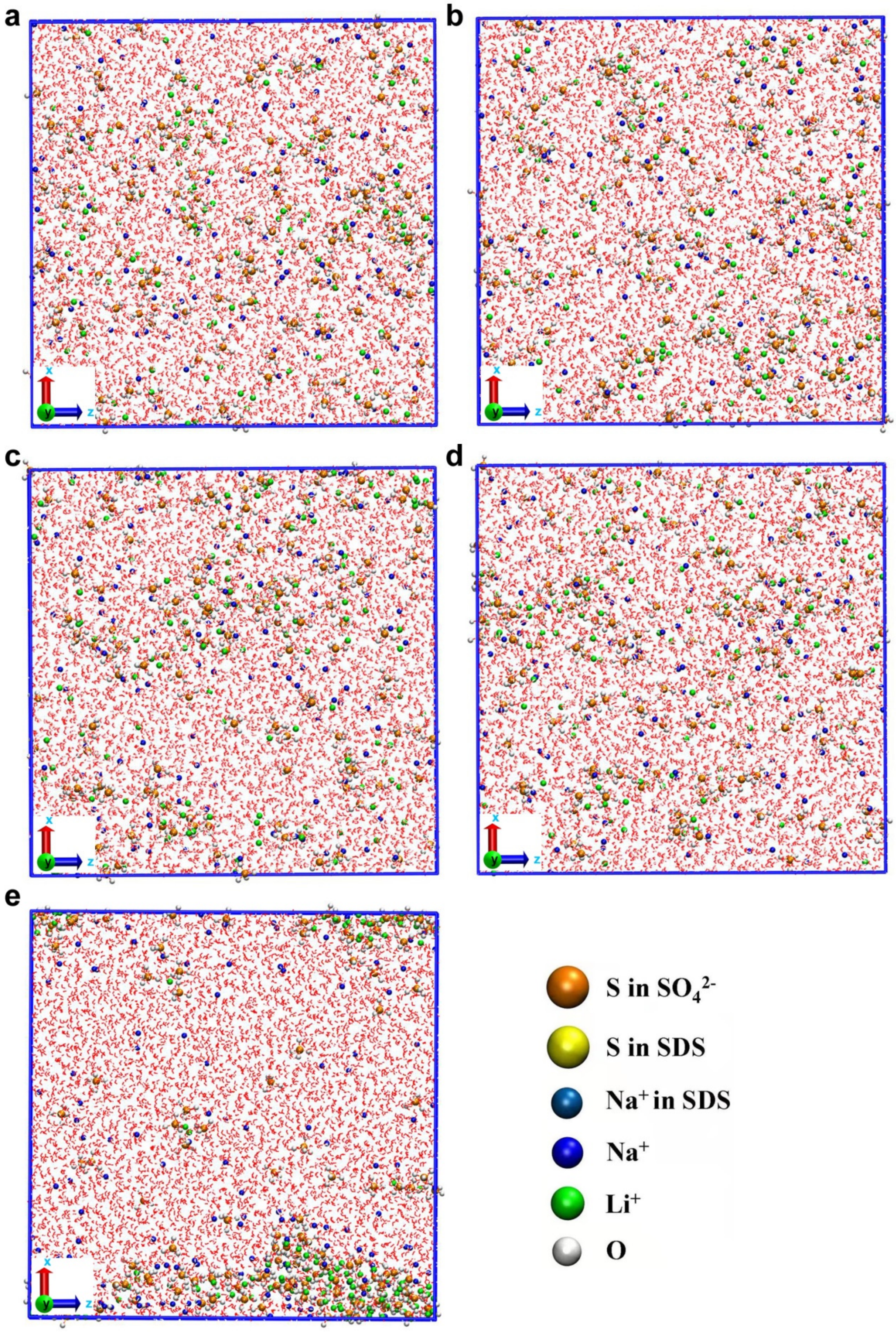

Figure S6. Molecular dynamic simulation results of LN-SDS-0 applying a voltage of 2 V nm^{-1} at: (**a**) 258.15 K, (**b**) 263.15 K, (**c**) 268.15 K, (**d**) 273.15 K, and (**e**) 298.15 K.

S2. Structural characterization of NTP and LMO

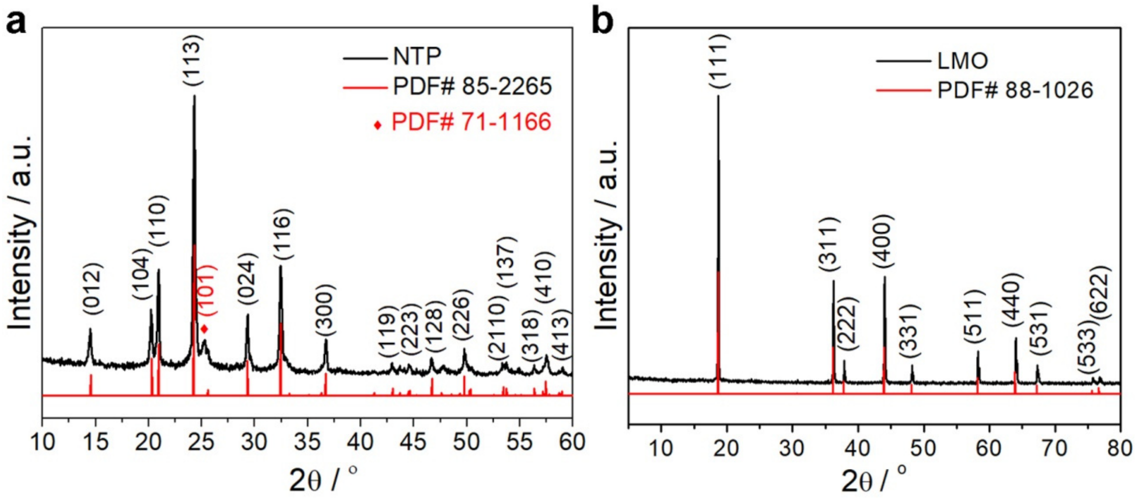

Figure S7. XRD patterns of (**a**) NTP and (**b**) LMO.

Figure S7 shows the XRD patterns of NTP and LMO. The diffraction peaks in Figure S7a match well with those of hexagonal NTP with the R-3c (167) space group (PDF# 85-2265), apart from a small peak centered at 25.2° corresponding to the (101) plane of anatase TiO_2 (PDF# 71-1166). The diffraction peaks in Figure S2 are in accordance with those of cubic LMO with the Fd-3m (227) space group (PDF# 88-1026).

The weight percent of TiO_2 in NTP can be calculated according to Equation (2), expressed as:[1]

$$\frac{I_1}{I_2} = \frac{K_1}{K_2}\frac{W_1}{W_2} \quad (2)$$

where I_i is the intensity of X-rays diffracted by a selected plane of component i, K_i is a constant which depends on the geometry of the diffractometer and the nature of component i, and W_i is the weight concentration of component i.

Using the XRD software of Jade 6 (MDI Jade 6.0, ICDD, CA, USA), the values of I_1 and I_2 were determined to be 100 and 3.8, corresponding to the diffraction peak intensity of (113) for NTP and of (101) for TiO_2, respectively. The values of K_1 and K_2 were determined to be 2.49 and 4.86 for NTP and TiO_2, respectively. On the premise that there is no other crystalline impurity in NTP, the weight percent of TiO_2 was estimated to be 1.91%.

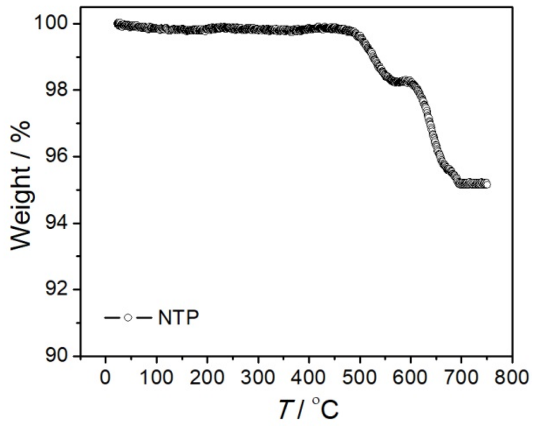

Figure S8. TG curve of NTP.

The weight percent of carbon in the NTP sample was determined through the TG analysis (Figure S8). It was found that the weight percent of carbon in the NTP sample was ca. 4.85 wt.%.

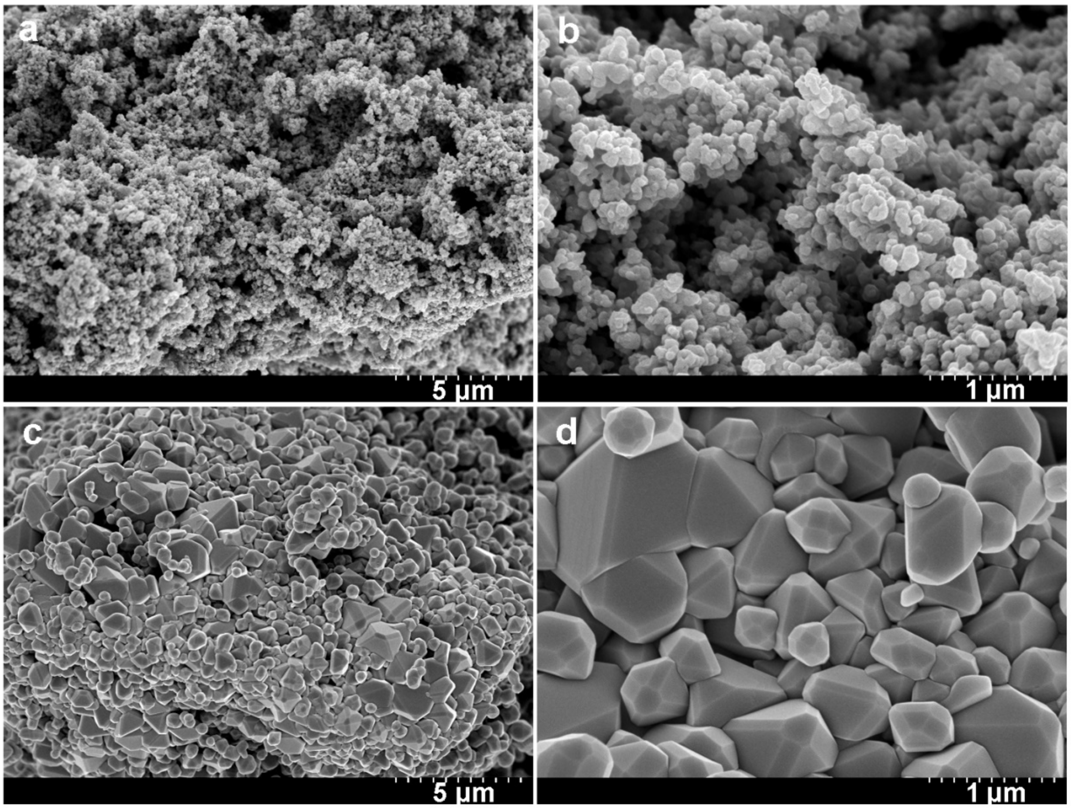

Figure S9. SEM images of (**a**,**b**) NTP and (**c**,**d**) LMO.

Figure S9 shows the SEM images of the samples. The NTP sample is composed of nanoparticles with a relatively narrow size distribution in the range of 60–120 nm (Figure S9a,b). LMO consists of polyhedral particles with size in the range of 0.15–1.5 μm and concentrating at 0.3–0.6 μm.

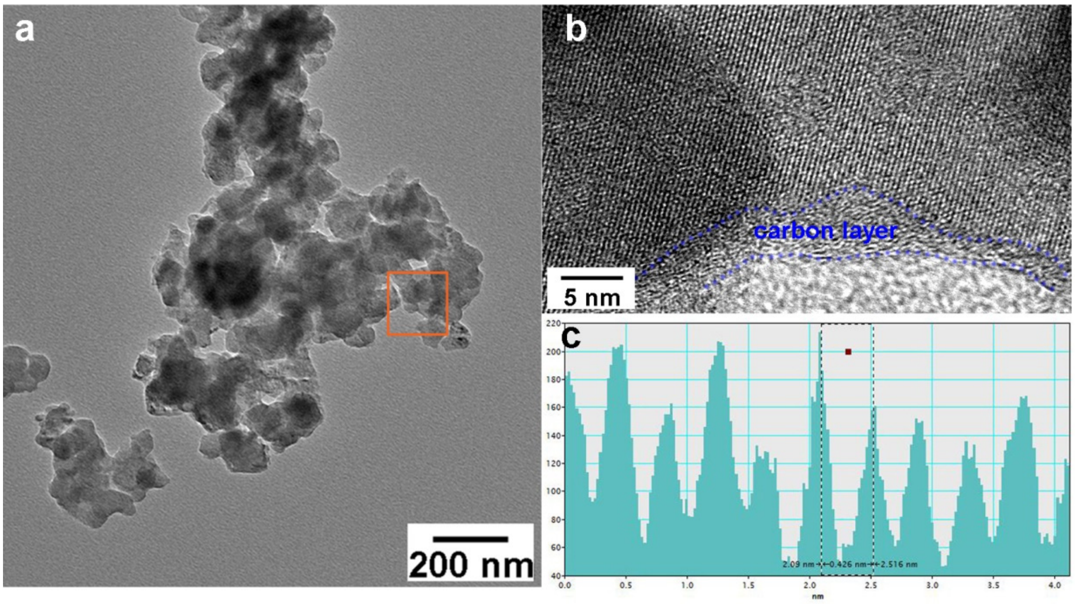

Figure S10. (**a**) TEM image, (**b**) high-resolution TEM image corresponding to the squared area in (a), and (**c**) lattice distance pattern obtained through the analysis with Gatan Software for NTP.

Figure S10 shows the TEM images of NTP. The carbon layer on the NTP surface can be discerned from the high-resolution TEM image, with thickness estimated to be ca. 2–6 nm (Figure S10b). The lattice fringes stop at the location where an amorphous carbon layer occurs. The distance between the lattice fringes was estimated to be 0.426 nm (Figure S10c), corresponding to the (110) plane of hexagonal NTP.

S3. Electrochemical performance in a three-electrode system

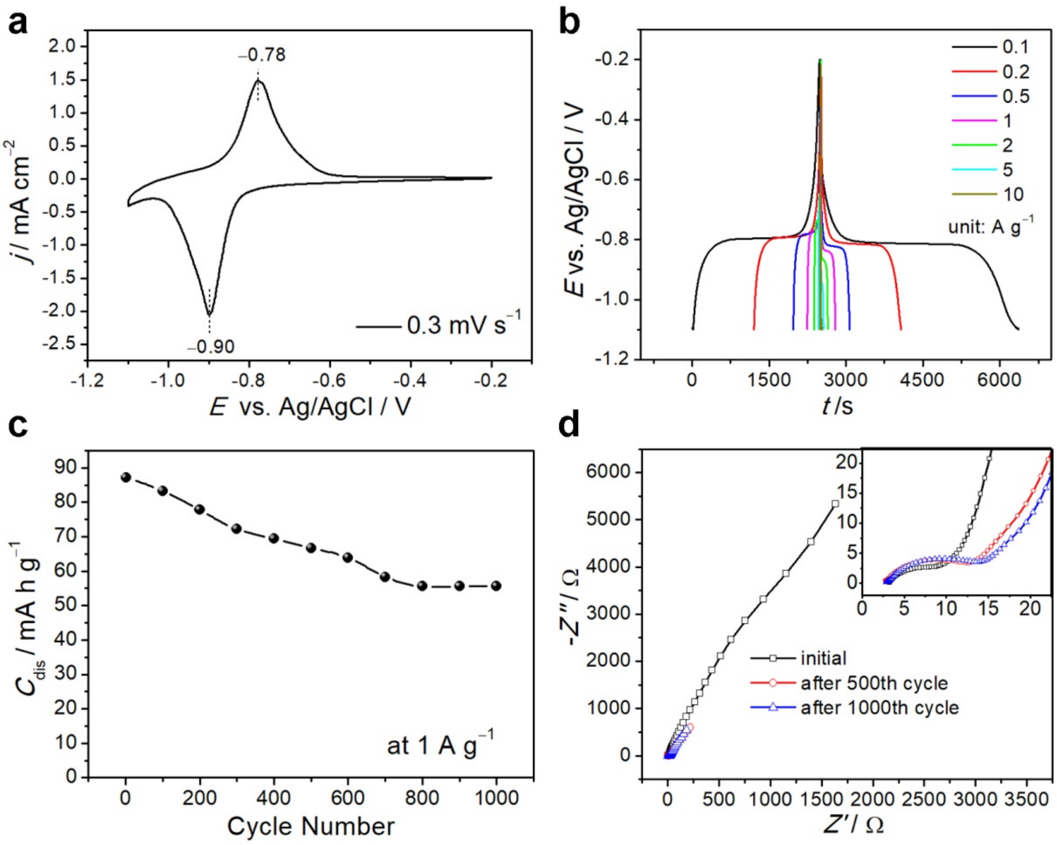

Figure S11. Electrochemical performance of NTP in a three-electrode system: (**a**) CV at 0.3 mV s^{-1} in a potential range of −(1.1–0.2) V, (**b**) GCD curves, (**c**) cycling performance at 1 A g^{-1}, and (**d**) Nyquist plots before and after cycling at 1 A g^{-1}.

The electrochemical performance of NTP was examined in a three-electrode system, using LN-SDS-0 (0.5 M Li_2SO_4 + 0.5 M Na_2SO_4) as the electrolyte. A pair of cathodic/anodic peaks centered at −0.90/−0.78 V occurred in the CV at a scan rate of 0.3 mV s^{-1} (Figure S11a), corresponding to the insertion/extraction of Na^+ in NTP.[2] Although Li^+ can also insert into NTP with a NASICON structure and Li^+ has a smaller ion radius relative to Na^+,[3] the Li^+ insertion is thermodynamically limited due to Li^+–Na^+ repulsion.[4] As a result, Na^+ ions preferably insert into NTP and occupy the M(1) sites in a Li^+/Na^+-mixed electrolyte.[4] At current densities of 0.1, 0.2, 0.5, 1, 2, 5, and 10 A g^{-1}, NTP exhibited the discharge-specific capacity (C_{dis}) of 108, 87, 79, 78, 74, 69, and 46 mA h g^{-1} in sequence (Figure S11b), comparable to that of NTP in our previous report using 1 M Na_2SO_4 as the electrolyte. [5] After cycling at 1 A g^{-1} for 1000 cycles, the C_{dis} remained at 56 mA h g^{-1}, presenting a capacity retention of 64.6% relative to the initial C_{dis} (Figure S11c). The capacity fading is attributed to NTP decomposition in aqueous electrolytes, [6,7] and side reactions of NTP with H_2O and O_2. [7,8] The capacity fading is also reflected by the Nyquist

plots (Figure S11d), where the charge transfer resistance increased from 6.8 to 11.5 and 12.8 Ω after 500 and 1000 cycles at 1 A g^{-1} in sequence.

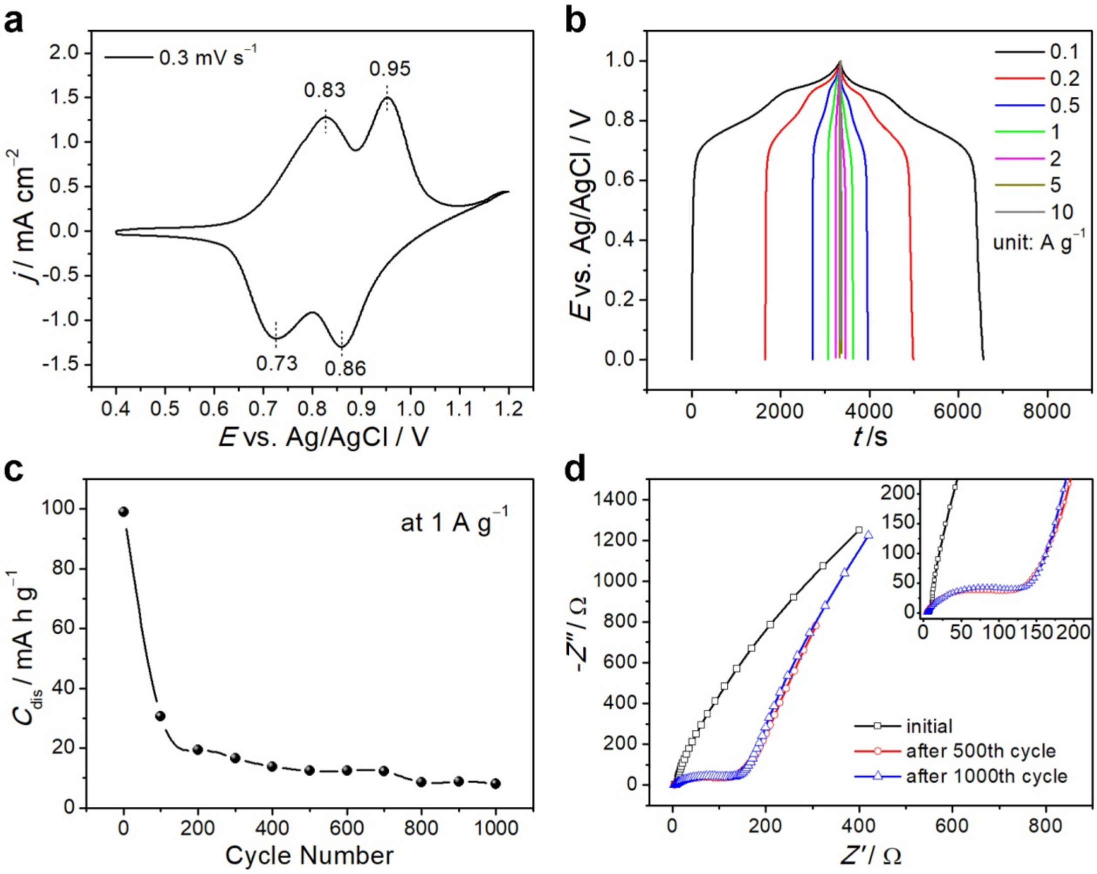

Figure S12. Electrochemical performance of LMO in a three-electrode system: (**a**) CV at 0.3 mV s^{-1} in a potential range of 0.4–1.2 V, (**b**) GCD curves, (**c**) cycling performance at 1 A g^{-1}, and (**d**) Nyquist plots before and after cycling at 1 A g^{-1}.

Figure S12 shows the electrochemical performance of LMO evaluated in a three-electrode system, using LN-SDS-0 as the electrolyte. Two pairs of cathodic/anodic peaks centered at 0.73/0.83 and 0.86/0.95 V occurred (Figure S12a), corresponding to two insertion/extraction steps of Li^+, rather than Na^+, into/from LMO, [9] as Na^+ with a relatively larger ion radius is difficult to insert into LMO. [4] The two-step insertion/extraction of Li^+ into/from LMO is also reflected by the GCD curves (Figure S12b), where two discharge/charge plateaus can be discerned. The C_{dis} values of LMO were 89, 91, 86, 77, 62, 33, and 6 mA h g^{-1} at current densities of 0.1, 0.2, 0.5, 1, 2, 5, and 10 A g^{-1}. After cycling at 1 A g^{-1} for 1000 cycles, the C_{dis} remained at 8 mA h g^{-1}, presenting an inferior cycling stability (Figure S12c). The capacity fading is attributed to the dissolution of LMO and side reactions of LMO with H_2O and trace O_2. [10,11] The capacity fading is also reflected by the Nyquist plots (Figure S12d), where the charge transfer resistance increased from 5.0 to 121.8 and 139.9 Ω after 500 and 1000 cycles at 1 A g^{-1} in sequence.

Based on the above analysis, it is apparent that the self-made NTP exhibited superior cycling stability in a three-electrode system, compared with the commercial LMO. The self-made NTP was then used as the anode to assemble NTP‖LMO full batteries.

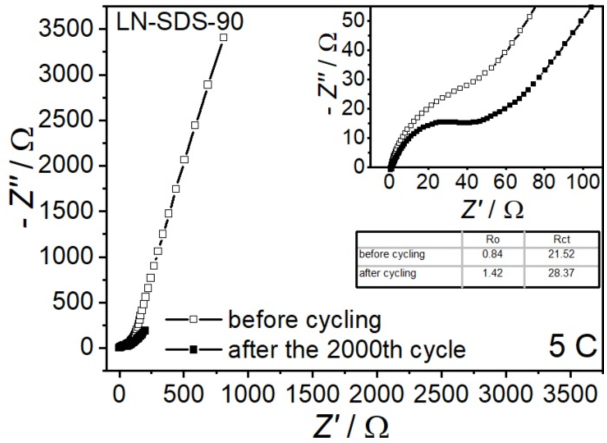

Figure S13. Nyquist plots of NTP‖LMO operating with LN-SDS-90 before and after cycling at 5 C for 2000 cycles, at room temperature.

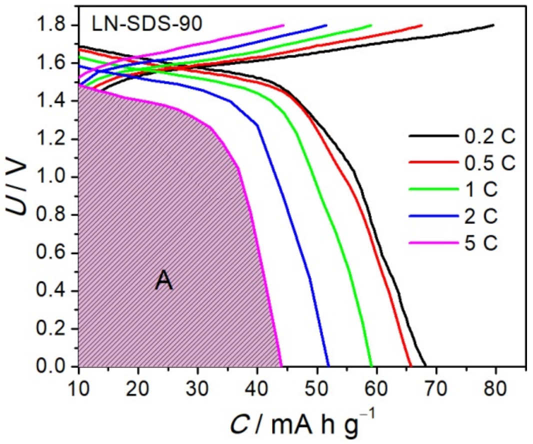

Figure S14. GCD curves of NTP‖LMO operating with LN-SDS-90 at varying C rates and at room temperature.

The average discharge potential (V_{ave}) of NTP‖LMO can be calculated according to Equation (3), expressed as (Reference [63] in the main text):

$$V_{ave} = A/C_{dis} \tag{3}$$

where A is the integrated area of the shadow, and C_{dis} is the discharge capacity.

The specific energy of a battery is obtained according to $E_{battery} = V_{ave} \times C_{dis}$.

Table S1. Comparison of the specific energy between NTP‖LMO and other ARMBs.

Battery	Electrolyte	E_{cell}(W h kg^{-1})/P_{cell}(W kg^{-1})/cycle number	Ref.
$NaTi_2(PO_4)_3/C \| Na_{0.44}MnO_2$	1 M Na_2SO_4	30/80/–*	[12]
$Na_2VTi(PO_4)_3/C \| Na_2VTi(PO_4)_3/C$	1 M Na_2SO_4	34/70/–	[13]
$NaTi_2(PO_4)_3/C \| Na_2NiFe(CN)_6$	1 M Na_2SO_4	42.5/130/–	[14]
$NaTi_2(PO_4)_3/C \| Na_{0.44}MnO_2$	2 M NaAc	20–30/50–1000/500	[15]
$AC \| LiMn_2O_4$	1 M Li_2SO_4	35/100/–	[16]

$NaTi_2(PO_4)_3/C \| LiMn_2O_4$	0.5 M Li_2SO_4 + 0.5 M Na_2SO_4	43/76.8/220	[9]
$NaTi_2(PO_4)_3/C \| LiMn_2O_4$	LN-SDS-90	41–28/90/2000	this work

* Data estimated from the information provided in the corresponding reference.

References

1. F. H. Chung,., Quantitative interpretation of X-ray diffraction patterns of mixtures. I. Matrix-flushing method for quantitative multicomponent analysis. *J. Appl. Cryst* 1974, **7**, 519-525.
2. L. Chen, L. Zhang, X. Zhou and Z. Liu, *ChemSusChem.*, Aqueous batteries based on mixed monovalence metal ions: a new battery family. 2014, **7**, 2295-2302.
3. L. Chen, J. Liu, Z. Guo, Y. Wang, C. Wang and Y. Xia, Electrochemical profile of $LiTi_2(PO_4)_3$ and $NaTi_2(PO_4)_3$ in lithium, sodium or mixed ion aqueous solutions. *J. Electrochem. Soc.*, 2016, **163**, A904-A910.
4. S. P. Ong, V. L. Chevrier, G. Hautier, A. Jain, C. Moore, S. Kim, X. Ma and G. Ceder, Voltage, stability and diffusion barrier differences between sodium-ion and lithium-ion intercalation materials. *Energy Environ. Sci.*, 2011, **4**, 3680.
5. X. Guo, Z. Luan, Y. Lu, L. Fu and L. Gai, $NaTi_2(PO_4)_3/C$ ‖ carbon package asymmetric flexible supercapacitors with the positive material recycled from spent Zn–Mn dry batteries. *J. Alloys Compd.*, 2019, **782**, 576-585.
6. S. I. Park, I. Gocheva, S. Okada and J.-i. Yamaki, Electrochemical properties of $NaTi_2(PO_4)_3$ anode for rechargeable aqueous sodium-ion batteries. *J. Electrochem. Soc.*, 2011, **158**, A1067.
7. A. I. Mohamed and J. F. Whitacre, Capacity fade of $NaTi_2(PO_4)_3$ in aqueous electrolyte solutions: relating pH increases to long term stability. *Electrochim. Acta* 2017, **235**, 730-739.
8. J. F. Whitacre, S. Shanbhag, A. Mohamed, A. Polonsky, K. Carlisle, J. Gulakowski, W. Wu, C. Smith, L. Cooney, D. Blackwood, J. C. Dandrea and C. Truchot, A Polyionic, Large–Format Energy Storage Device Using an Aqueous Electrolyte and Thick–Format Composite $NaTi_2(PO_4)_3$/Activated Carbon Negative Electrodes. *Energy Technol.*, 2015, 20-31.
9. Y. Kong, J. Sun, L. Gai, X. Ma and J. Zhou, $NaTi_2(PO_4)_3/C$ ‖ $LiMn_2O_4$ rechargeable battery operating with Li^+/Na^+-mixed aqueous electrolyte exhibits superior electrochemical performance. *Electrochimica Acta*, 2017, **255**, 220-229.
10. J. Y. Luo, W. J. Cui, P. He and Y. Y. Xia, Raising the cycling stability of aqueous lithium-ion batteries by eliminating oxygen in the electrolyte.. *Nat. Chem.*, 2010, **2**, 760-765.
11. D. M. Robinson, Y. B. Go, M. Greenblatt and G. C. Dismukes, Water Oxidation by λ-MnO_2: Catalysis by the Cubical Mn_4O_4 Subcluster Obtained by Delithiation of Spinel $LiMn_2O_4$. *J. Am. Chem. Soc.*, 2010, **132**, 11467–11469.
12. Z. Li, D. Young, K. Xiang, W. C. Carter and Y.-M. Chiang, Towards High Power High Energy Aqueous Sodium–Ion Batteries: The $NaTi_2(PO_4)_3/Na_{0.44}MnO_2$ System. *Adv. Energy Mater.*, 2013, **3**, 290-294.
13. J. Dong, G. Zhang, X. Wang, S. Zhang and C. Deng, Cross-linked $Na_2VTi(PO_4)_3$@C hierarchical nanofibers as high-performance bi-functional electrodes for symmetric aqueous rechargeable sodium batteries. *J. Mater. Chem. A*, 2017, **5**, 18725-18736.
14. X. Wu, Y. Cao, X. Ai, J. Qian and H. Yang, A low-cost and environmentally benign aqueous rechargeable sodium-ion battery based on $NaTi_2(PO_4)_3$–$Na_2NiFe(CN)_6$ intercalation chemistry. *Electrochem. Commun.*, 2013, **31**, 145-148.
15. Z. Hou, X. Li, J. Liang, Y. Zhu and Y. Qian, An aqueous rechargeable sodium ion battery based on a $NaMnO_2$–$NaTi_2(PO_4)_3$ hybrid system for stationary energy storage. *J. Mater. Chem. A*, 2015, **3**, 1400-1404.
16. Y.-g. Wang and Y.-y. Xia, Hybrid Aqueous Energy Storage Cells Using Activated Carbon and Lithium-Intercalated Compounds: I. The System. *J. Electrochem. Soc.*, 2006, **153**, A450.
